# Condom and Contraceptive Use Among Sexually Active High School Students — Youth Risk Behavior Survey, United States, 2019

**DOI:** 10.15585/mmwr.su6901a2

**Published:** 2020-08-21

**Authors:** Leigh E. Szucs, Richard Lowry, Amy M. Fasula, Sanjana Pampati, Casey E. Copen, Khaleel S. Hussaini, Rachel E. Kachur, Emilia H. Koumans, Riley J. Steiner

**Affiliations:** ^1^Division of Adolescent and School Health, National Center for HIV/AIDS, Viral Hepatitis, STD, and TB Prevention, CDC; ^2^Office of the Director, National Center for HIV/AIDS, Viral Hepatitis, STD, and TB Prevention, CDC; ^3^Division of Reproductive Health, National Center for Chronic Disease Prevention and Health Promotion, CDC; ^4^Oak Ridge Institute for Science and Education; ^5^Division of STD Prevention, National Center for HIV/AIDS, Viral Hepatitis, STD, and TB Prevention CDC

## Abstract

Preventing unintended pregnancy and sexually transmitted diseases (STDs), including human immunodeficiency virus (HIV) infection, among adolescents is a public health priority. This report presents prevalence estimates for condom and contraceptive use among sexually active U.S. high school students from the 2019 Youth Risk Behavior Survey. Behaviors examined included any condom use, primary contraceptive method use, and condom use with a more effective contraceptive method, all reported at last sexual intercourse. Analyses were limited to sexually active students (i.e., those who had sexual intercourse with one or more persons during the 3 months before the survey). Except for any condom use, students reporting only same-sex sexual contact were excluded from analyses. Weighted prevalence estimates were calculated, and bivariate differences in prevalence were examined by demographic characteristics (sex, race/ethnicity, and grade) and other sexual risk behaviors (age of sexual initiation, previous 3-month and lifetime number of sex partners, and substance use before last sexual intercourse). Nationwide, 27.4% of high school students reported being sexually active (n = 3,226). Among sexually active students who reported having had sexual contact with someone of the opposite sex (n = 2,698), most students (89.7%) had used a condom or a primary contraceptive method at last sexual intercourse. Prevalence of any condom use at last sexual intercourse was 54.3%, and condoms were the most prevalent primary contraceptive method (43.9% versus 23.3% for birth control pills; 4.8% for intrauterine device [IUD] or implant; and 3.3% for shot, patch, or ring). Approximately 9% had used condoms with an IUD, implant, shot, patch, ring, or birth control pills. Using no pregnancy prevention method was more common among non-Hispanic black (23.2%) and Hispanic (12.8%) students compared with non-Hispanic white students (6.8%); compared with Hispanic students, using no pregnancy prevention method was more common among non-Hispanic black students. Prevalence of condom use was consistently lower among students with other sexual risk behaviors. Results underscore the need for public health professionals to provide quality sexual and reproductive health education and clinical services for preventing unintended pregnancy and STDs/HIV and decreasing disparities among sexually active youths.

## Introduction

Preventing unintended pregnancy and sexually transmitted diseases (STDs), including human immunodeficiency virus (HIV) infection, is a U.S. public health priority, particularly among adolescents (*1*). U.S. birth rates among youths aged 15–19 years have decreased to record lows; evidence suggests that increasing use of a range of contraceptive options, including intrauterine devices (IUDs) and implants, also known as long-acting reversible contraception, is a contributing factor (*2*). However, U.S. birth rates among adolescents remain higher than rates in comparable Western industrialized nations (*3*). In 2018, U.S. birth rates for persons aged 15–17 and 18–19 years were 7.2 and 32.3 births per 1,000 females, respectively (*4*). Moreover, racial/ethnic, geographic, and socioeconomic disparities persist (*4*). For example, in 2018, birth rates among non-Hispanic black (black) (26.3) and Hispanic (26.7) persons aged 15–19 years were almost two times the rate for non-Hispanic white (white) (12.1) persons (*4*). 

Contraceptive methods vary in effectiveness and highly and moderately effective methods do not prevent STDs, which disproportionately affect adolescents (*5*). Highly effective reversible contraceptive methods (IUDs and implants) are associated with a <1% failure rate during the first year of typical use; moderately effective contraceptive methods (injectables, patches, rings, and birth control pills) are associated with a 4%–7% failure rate during the first year of typical use; and less effective methods (condoms, diaphragm, and spermicides) are associated with a >10% failure rate during the first year of typical use (*6*). Condoms, although categorized as a less effective method of pregnancy prevention (*6*), remain vital for STD/HIV prevention and promoting condom use is particularly important given increasing STD rates in the United States (*5*). Professional medical organizations (*7*,*8*) and federal agencies, including CDC, recommend using condoms for STD/HIV prevention with a more effective method of contraception for optimal protection against unintended pregnancy (*9*). However, recent decreases in condom use have been documented, and the proportion of adolescents using condoms with more effective methods of contraception has been consistently low, with recent national estimates of approximately 9% of sexually active high school students (*10*).

Because of these challenges to pregnancy- and STD/HIV-prevention goals, monitoring condom and contraceptive use behaviors among sexually active youths is essential. This study reports prevalence estimates from the 2019 Youth Risk Behavior Survey (YRBS) for any condom use at last sexual intercourse among sexually active U.S. high school students. In addition, prevalence estimates of primary contraceptive method use and condom use with more effective methods of contraception at last sexual intercourse among sexually active students who had sexual contact with the opposite sex during their lifetime are reported. Variations in these behaviors by demographic characteristics and sexual risk behaviors were examined to support public health professionals in implementing quality sexual and reproductive health education and clinical services that prevent STDs/HIV and unintended pregnancy.

## Methods

### Data Source

This report includes data from the 2019 YRBS, a cross-sectional, school-based survey conducted biennially since 1991. Each survey year, CDC collects data from a nationally representative sample of public and private school students in grades 9–12 in the 50 U.S. states and the District of Columbia. Additional information about YRBS sampling, data collection, response rates, and processing is available in the overview report of this supplement (*11*). The prevalence estimates for all sexual behavior questions for the overall study population and by sex, race/ethnicity, grade, and sexual orientation are available at https://nccd.cdc.gov/youthonline/App/Default.aspx. The full YRBS questionnaire is available at https://www.cdc.gov/healthyyouth/data/yrbs/pdf/2019/2019_YRBS-National-HS-Questionnaire.pdf.

### Measures

Behaviors analyzed included any condom use, primary contraceptive method, and condom use with more effective methods of contraception, all reported at last sexual intercourse. Any condom use was assessed by the question, “The last time you had sexual intercourse, did you or your partner use a condom?” Response options included the following: I have never had sexual intercourse, yes, or no. Primary contraceptive method was assessed through a separate question, “The last time you had sexual intercourse, what one method did you or your partner use to prevent pregnancy?” Respondents could select only one response from the following list of options: I have never had sexual intercourse; no method was used to prevent pregnancy; birth control pills; condoms; an IUD (such as Mirena or ParaGard) or implant (such as Implanon or Nexplanon); a shot (such as Depo-Provera), patch (such as Ortho Evra), or birth control ring (such as NuvaRing); withdrawal or some other method; or not sure. Dichotomous (yes versus no) variables for each response option were created, except for “not sure”; although participants selecting this response (n = 93; 3.9%) were included in the analytic sample, prevalence estimates for this category are not reported.

A dichotomous (yes versus no) variable for any condom use with an IUD, implant, shot, patch, ring, or birth control pills was constructed by using the separate items for any condom use and primary contraceptive method at last sexual intercourse. These two items were also used to create the following dichotomous (yes versus no) indicators: condom use only (yes to any condom use and condoms or no method for pregnancy prevention); highly or moderately effective contraceptive use only (no to any condom use and an IUD, implant, shot, patch, ring, or birth control pills for pregnancy prevention); withdrawal or some other contraceptive method use only (no to any condom use and withdrawal or some other method for pregnancy prevention); and use of no condom and no primary contraceptive method (no to any condom use and no method for pregnancy prevention).

Condom and contraceptive use were examined by demographic characteristics and sexual risk behaviors. Demographic characteristics included sex (female or male), race/ethnicity (non-Hispanic white [white], non-Hispanic black [black], or Hispanic; other/multiple responses are not reported), and grade (9, 10, 11, or 12). Four dichotomous sexual risk behaviors were created: age of sexual initiation (<13 years versus ≥13 years); lifetime number of sex partners (1–3 versus ≥4); number of sex partners during the previous 3 months (1 versus ≥2); and alcohol or drug use before last sexual intercourse (yes versus no).

### Analysis

The analytic sample was restricted to sexually active students (i.e., those who reported having had sexual intercourse with one or more persons during the 3 months before the survey). Analyses involving pregnancy prevention methods excluded students who only had same-sex sexual contacts during their lifetime, on the basis of an item about respondents’ sex (“What is your sex?” with response options including female or male) and another item assessing the sex of sexual contacts (“During your life, with whom have you had sexual contact?” with response options including I have never had sexual contact, females, males, and females and males).

 All analyses were conducted using SUDAAN (version 11.0.0; RTI International) to account for the complex sampling design. Weighted prevalence estimates and 95% confidence intervals were calculated for each outcome. Chi-square statistics were used to examine bivariate differences by demographic characteristics and sexual risk behaviors. For significant overall differences by race/ethnicity and grade, *t*-tests were used to identify pairwise differences. Differences were considered significant if p<0.05.

## Results

Among the 27.4% of sexually active students (n = 3,226), approximately half were female (52.2%) and white (52.3%); approximately one third were in grade 12 (36.9%) (Table 1). Regarding sexual risk behaviors among those sexually active students, 7.0% had sexual intercourse for the first time before age 13 years (3.0% of all YRBS respondents reported having had sexual intercourse for the first time before age 13 years); 26.9% had sexual intercourse with ≥4 persons during their lifetime (8.6% of all YRBS respondents reported having had sexual intercourse with ≥4 persons during their lifetime); 20.5% had sexual intercourse with ≥2 persons during the previous 3 months; and 21.2% had drunk alcohol or used drugs before last sexual intercourse.

**TABLE 1:** Prevalence of demographic characteristics and sexual risk behaviors among sexually active* high school students — Youth Risk Behavior Survey, United States, 2019

Characteristic	No.^†^ (%^§^)	95% CI
**Sex**		
Female	1,679 (52.2)	49.4–55.0
Male	1,510 (47.8)	45.0–50.6
**Race/Ethnicity^¶^**		
Black, non-Hispanic	474 (11.2)	8.9–14.0
Hispanic	771 (28.4)	22.3–35.5
White, non-Hispanic	1,602 (52.3)	46.4–58.1
**Grade**		
9	389 (11.3)	9.8–13.0
10	741 (21.4)	19.3–23.6
11	967 (30.4)	27.8–33.2
12	1,089 (36.9)	33.4–40.4
**Sexual risk behavior**		
Had sexual intercourse before age 13 years	242 (7.0)	5.7–8.5
Had sexual intercourse with ≥4 persons during their lifetime	854 (26.9)	24.3–29.7
Had sexual intercourse with ≥2 persons during the previous 3 months	658 (20.5)	18.5–22.7
Had drunk alcohol or used drugs before last sexual intercourse	652 (21.2)	18.8–23.9

**Abbreviation:** CI = confidence interval.

* Defined as having had sexual intercourse with at least one person during the 3 months before the survey (n = 3,226).

^†^ Unweighted.

^§^ Weighted estimates.

^¶^ Race/ethnicity values do not total 100% because “other/multiple” responses are not reported (i.e., American Indian/Alaska Native, Asian, Native Hawaiian/Other Pacific Islander, and multiple race).

Among sexually active students, prevalence of any condom use at last sexual intercourse was 54.3% (Table 2). Among sexually active students who reported having had sexual contact with someone of the opposite sex (i.e., excluding those who reported only same-sex sexual contact) (n = 2,698), condoms (43.9%) were the most prevalent primary pregnancy prevention method, based on responses to the distinct item assessing pregnancy prevention method, followed by birth control pills (23.3%); withdrawal or other method (10.1%); IUD or implant (4.8%); and shot, patch, or ring (3.3%). (Of participants excluded from the analytic sample for primary method of pregnancy prevention, 95 students reported having had only same-sex sexual contact and 433 students did not answer the questions, “What is your sex?” or “During your life, with whom have you had sexual contact?”) Approximately one tenth (10.7%) had not used a pregnancy prevention method at last sexual intercourse; 9.1% had used a condom with an IUD, implant, shot, patch, ring, or birth control pills at last sexual intercourse. Prevalence of condom and IUD or implant use (<1.0%) and condom and shot, patch, or ring use (<1.0%) was lower than condom and birth control pills use (7.5%).

**TABLE 2:** Prevalence of condom and primary contraceptive use at last sexual intercourse among sexually active* high school students, by demographic characteristics — Youth Risk Behavior Survey, United States, 2019

Demographic characteristic	Any condom use^†^		Primary contraceptive method												Condoms and IUD, implant, shot, patch, ring, or birth control pills	
			IUD or implant		Shot, patch, or ring		Birth control pills		Condom		Withdrawal or other method		No method			
	%^§^ (95% CI)	p value^¶^	%^§^ (95% CI)	p value^¶^	%^§^ (95% CI)	p value^¶^	%^§^ (95% CI)	p value^¶^	%^§^ (95% CI)	p value^¶^	%^§^ (95% CI)	p value^¶^	%^§^ (95% CI)	p value^¶^	%^§^ (95% CI)	p value^¶^
**Total**	**54.3 (52.0–56.6)**	**NA**	**4.8 (3.3–7.0)**	**NA**	**3.3 (2.3–4.7)**	**NA**	**23.3 (19.8–27.2)**	**NA**	**43.9 (40.6–47.3)**	**NA**	**10.1 (8.5–12.0)**	**NA**	**10.7 (8.8–12.8)**	**NA**	**9.1 (7.4–11.2)**	**NA**
**Sex**	NA	<0.01	NA	0.20	NA	<0.05	NA	<0.01	NA	<0.01	NA	0.34	NA	0.21	NA	0.10
Female	49.6 (45.6–53.6)	NA	5.6 (4.0–7.6)	NA	4.5 (2.9–6.8)	NA	26.1 (22.1–30.5)	NA	38.8 (34.0–44.0)	NA	10.8 (8.9–13.0)	NA	11.9 (9.1–15.3)	NA	10.3 (8.3–12.7)	NA
Male	60.0 (57.0–63.0)	NA	4.0 (2.1–7.4)	NA	2.1 (1.2–3.6)	NA	20.2 (16.4–24.7)	NA	49.4 (45.8–53.1)	NA	9.3 (7.1–12.2)	NA	9.3 (7.1–12.1)	NA	7.9 (5.8–10.7)	NA
**Grade**	NA	<0.05	NA	<0.01	NA	0.29	NA	<0.01	NA	<0.01	NA	0.20	NA	0.63	NA	<0.01
9	61.3** (54.6–67.5)	NA	0.1**^,††,§§^ (0.0–0.7)	NA	2.4 (0.9–6.2)	NA	10.9**^,††^ (6.0–19.1)	NA	55.3**^,††^ (47.4–62.9)	NA	10.6 (6.9–15.9)	NA	14.1 (9.1–21.2)	NA	4.7** (2.7–8.2)	NA
10	55.4 (50.2–60.4)	NA	3.3** (2.0–5.6)	NA	2.1 (1.0–4.4)	NA	18.2**^,††^ (13.4–24.3)	NA	47.7** (41.2–54.3)	NA	12.5 (9.5–16.4)	NA	10.5 (7.2–15.1)	NA	7.0** (4.7–10.3)	NA
11	56.3 (51.9–60.6)	NA	3.2** (1.8–5.8)	NA	4.2 (2.6–6.7)	NA	25.8 (21.0–31.3)	NA	45.3** (39.4–51.4)	NA	7.9 (5.9–10.5)	NA	10.1 (7.9–12.8)	NA	8.9 (6.1–12.9)	NA
12	50.3 (46.9–53.8)	NA	8.2 (5.5–12.2)	NA	3.6 (2.0–6.3)	NA	27.7 (23.3–32.5)	NA	37.4 (33.4–41.6)	NA	10.3 (7.9–13.3)	NA	10.2 (6.9–14.9)	NA	11.6 (8.9–15.0)	NA
**Race/Ethnicity**	NA	<0.05	NA	<0.01	NA	0.07	NA	<0.01	NA	<0.01	NA	<0.01	NA	<0.01	NA	<0.01
Black, non-Hispanic	48.2^¶¶,^*** (43.2–53.3)	NA	2.0^¶¶^ (1.0–4.0)	NA	5.4 (2.9–9.9)	NA	12.1^¶¶^ (8.7–16.5)	NA	37.2*** (31.2–43.6)	NA	13.9^¶¶^ (8.4–22.2)	NA	23.2^¶¶,^*** (19.2–27.7)	NA	7.5^¶¶^ (5.1–10.8)	NA
Hispanic	56.2 (52.0–60.3)	NA	1.6^¶¶^ (0.7–3.4)	NA	1.4 (0.6–3.2)	NA	15.5^¶¶^ (11.5–20.5)	NA	49.6^¶¶^ (44.7–54.4)	NA	13.1^¶¶^ (10.0–17.0)	NA	12.8^¶¶^ (9.1–17.8)	NA	4.8^¶¶^ (3.1–7.4)	NA
White, non-Hispanic	55.8 (52.9–58.6)	NA	6.7 (5.0–9.0)	NA	4.0 (2.5–6.4)	NA	29.7 (25.7–34.0)	NA	42.3 (38.2–46.5)	NA	7.7 (6.1–9.8)	NA	6.8 (5.3–8.6)	NA	12.4 (10.1–15.2)	NA

**Abbreviations:** CI = confidence interval; IUD = intrauterine device; NA = not applicable.

* Defined as having had sexual intercourse with at least one person during the 3 months before the survey (n = 3,226). Except for any condom use at last sexual intercourse, students reporting only same-sex sexual contact use were excluded; therefore, the analytic sample was restricted to sexually active students who reported having had sexual contact with someone of the opposite sex (n = 2,698). Among sexually active students, excluding those who only had same-sex sexual contact, a total of 93 (3.9%) students answered the pregnancy prevention question “not sure”; findings are not presented for this group.

^†^ Any condom use at last sexual intercourse was measured by a separate item from condoms as the primary method used for preventing pregnancy.

^§^ Weighted estimates.

^¶^ Significance is defined as p<0.05, by chi-square test.

** Significantly different than grade 12, by linear contrast *t*-test

^††^ Significantly different than grade 11, by linear contrast *t*-test.

^§§^ Significantly different than grade 10, by linear contrast *t*-test.

^¶¶^ Significantly different than white, non-Hispanic race/ethnicity, by linear contrast *t*-test.

*** Significantly different than Hispanic race/ethnicity, by linear contrast *t*-test.

Prevalence estimates for mutually exclusive categories that reflect both pregnancy and STD/HIV prevention effectiveness and account for any condom use in addition to a primary pregnancy prevention method indicate that condom use only was most common (44.3%), followed by highly or moderately effective contraceptive method use only (22.2%) (Figure). Prevalence of condom use with an IUD, implant, shot, patch, ring, or birth control pills (9.1%) was similar to prevalence of using withdrawal or other method only (9.5%) and using no condom and no primary pregnancy prevention method (10.3%).

**FIGURE:**
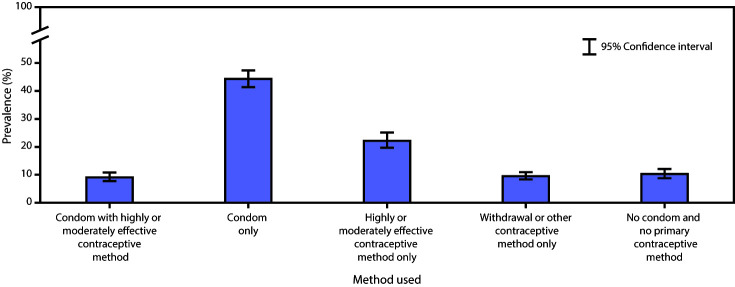
Prevalence of condom and primary contraceptive use* at last sexual intercourse among sexually active^†^ high school students — Youth Risk Behavior Survey, United States, 2019 * **Condom with highly or moderately effective contraceptive method** = students who responded “yes” to any condom use at last sexual intercourse and intrauterine device or implant; shot, patch, or ring; or birth control pills (i.e., highly or moderately effective methods) as primary pregnancy prevention method**. Condom only** = students who responded “yes” to any condom use at last sexual intercourse and condom or no method as primary pregnancy prevention method. **Highly or moderately effective contraceptive method only** = students who responded “no” to any condom use at last sexual intercourse and intrauterine device or implant; shot, patch, or ring; or birth control pills (i.e., highly or moderately effective methods) as primary pregnancy prevention method. **Withdrawal or other contraceptive method only** = students who responded “no” to any condom use at last sexual intercourse and withdrawal or some other method as primary pregnancy prevention method. **No condom and no primary contraceptive method** = students who responded “no” to any condom use at last sexual intercourse and no method of pregnancy prevention. ^†^ Defined as having had sexual intercourse with at least one person during the 3 months before the survey (n = 2,698). Students reporting only same-sex sexual contact were excluded from the analytic sample.

Analyses revealed significant differences in any condom use and primary pregnancy prevention method at last sexual intercourse by demographic characteristics (Table 2). By sex, no differences occurred in not using any method of pregnancy prevention (i.e., no method); however, differences were identified in type of method used. Compared with male students’ report of contraceptive use by their female partner, prevalence as reported by female students was higher for shot, patch, or ring (4.5% versus 2.1%) and birth control pills (26.1% versus 20.2%). In contrast, prevalence of condom use as the primary method for pregnancy prevention reported by male students (49.4%) was higher than female students’ report of condom use by their male partner (38.8%), as was any condom use at last sexual intercourse (60.0% versus 49.6%).

A similar pattern emerged when examining prevalence of any condom and primary contraceptive method use by grade. The prevalence of using no method was the same across grades; however, differences occurred in method type. Any condom use and condom use as the primary pregnancy prevention method was more prevalent in lower versus higher grades. In contrast, use of an IUD or implant; birth control pills; and condom with an IUD or implant, shot, patch, ring, or birth control pills was typically more prevalent in higher versus lower grades. For example, condom use as the primary pregnancy prevention method was more common among students in grades 9 (55.3%), 10 (47.7%), and 11 (45.3%) versus students in grade 12 (37.4%) (and grade 9 versus grade 11), whereas IUD or implant use was less common among 9th-grade students (<1.0%), compared with students in grades 10 (3.3%), 11 (3.2%), and 12 (8.2%). Prevalence of IUD or implant use among 10th- and 11th-grade students was also lower than among 12th-grade students.

In contrast with sex and grade, not using a pregnancy prevention method differed by race/ethnicity, with higher prevalence of no method among black (23.2%) and Hispanic (12.8%) students, compared with white students (6.8%); compared with Hispanic students, using no pregnancy prevention method was more common among black students. Additional racial/ethnic differences in type of method were identified, with the general pattern that prevalence of using a more effective method of contraception was lower among black and Hispanic students compared with white students. Specifically, prevalence among black and Hispanic students was lower than among white students for use of an IUD or implant (2.0% and 1.6% versus 6.7%, respectively); birth control pills (12.1% and 15.5% versus 29.7%, respectively); and condom use with an IUD, implant, shot, patch, ring, or birth control pills (7.5% and 4.8% versus 12.4%, respectively). In contrast, prevalence of withdrawal or other method use was higher among black (13.9%) and Hispanic (13.1%) students than among white students (7.7%). Condom use as the primary method for pregnancy prevention was higher among Hispanic students (49.6%), compared with black (37.2%) and white (42.3%) students, and any condom use at last sexual intercourse was higher among Hispanic (56.2%) and white (55.8%) students compared with black students (48.2%).

Differences by sexual risk behaviors in the prevalence of using no contraceptive method and in the type of method used, including any condom use, also were observed (Table 3). Comparing students who had initiated sex before age 13 years with students who had not, differences in no method use were not significant; however, prevalence was lower for any condom use at last sexual intercourse (40.9% versus 55.4%), condom use as the primary method of pregnancy prevention (30.4% versus 44.8%), and withdrawal or other method use (5.5% versus 10.4%). Students who had ≥4 lifetime partners had higher prevalence of no method use (14.7% versus 9.2%) and lower prevalence of any condom use (46.6% versus 57.1%); condom use as the primary pregnancy prevention method (36.2% versus 46.6%); and condom use with an IUD, implant, shot, patch, ring, or birth control pills (6.5% versus 10.1%) compared with students who had <4 lifetime sex partners. A similar pattern was observed for students who reported having had ≥2 recent partners, although no significant differences in no method use were observed. Comparing students who had drunk alcohol or used drugs before last sexual intercourse with students who had not, use of no method was higher (14.7% versus 9.6%), whereas any condom use (47.4% versus 56.0%) and condom use as the primary pregnancy prevention method (39.3% versus 45.1%) were lower.

**TABLE 3:** Prevalence of condom and primary contraceptive use at last sexual intercourse among sexually active* high school students, by sexual risk behaviors — Youth Risk Behavior Survey, United States, 2019

Sexual risk behavior	Any condom use^†^		Primary contraceptive method												Condom and IUD, implant, shot, patch, ring, or birth control pills	
			IUD or implant		Shot, patch, or ring		Birth control pills		Condom		Withdrawal or other method		No method			
	%^§^ (95% CI)	p value^¶^	%^§^ (95% CI)	p value^¶^	%^§^ (95% CI)	p value^¶^	%^§^ (95% CI)	p value^¶^	%^§^ (95% CI)	p value^¶^	%^§^ (95% CI)	p value^¶^	%^§^ (95% CI)	p value^¶^	%^§^ (95% CI)	p value^¶^
**Had sexual intercourse before age 13 years**	NA	<0.05	NA	0.05	NA	0.83	NA	0.81	NA	<0.05	NA	<0.05	NA	0.05	NA	0.12
Yes	40.9 (30.4–52.4)	NA	2.6 (1.1–6.0)	NA	3.0 (1.0–8.2)	NA	22.1 (13.4–34.1)	NA	30.4 (19.3–44.2)	NA	5.5 (2.9–10.3)	NA	22.8 (12.6–37.7)	NA	4.8 (1.7–13.3)	NA
No	55.4 (52.9–57.9)	NA	5.0 (3.4–7.2)	NA	3.4 (2.3–4.9)	NA	23.4 (19.7–27.5)	NA	44.8 (41.4–48.3)	NA	10.4 (8.6–12.5)	NA	9.8 (8.0–12.0)	NA	9.4 (7.6–11.6)	NA
**Had sexual intercourse with ≥4 persons during their lifetime**	NA	<0.01	NA	<0.01	NA	0.60	NA	0.43	NA	<0.01	NA	0.32	NA	<0.05	NA	<0.05
Yes	46.6 (42.9–50.2)	NA	7.4 (5.0–10.8)	NA	2.9 (1.6–5.2)	NA	21.5 (17.0–26.9)	NA	36.2 (31.0–41.7)	NA	11.5 (8.7–15.0)	NA	14.7 (10.9–19.6)	NA	6.5 (4.3–9.8)	NA
No	57.1 (54.3–59.8)	NA	3.9 (2.6–5.8)	NA	3.5 (2.3–5.4)	NA	23.9 (19.7–28.6)	NA	46.6 (43.1–50.1)	NA	9.6 (7.7–11.9)	NA	9.2 (7.3–11.7)	NA	10.1 (8.1–12.4)	NA
**Had sexual intercourse with ≥2 persons during the previous 3 months**	NA	<0.01	NA	0.69	NA	<0.05	NA	0.13	NA	<0.05	NA	0.14	NA	0.20	NA	<0.01
Yes	47.1 (43.1–51.1)	NA	5.0 (3.1–8.0)	NA	1.7 (0.6–4.5)	NA	19.9 (15.1–25.8)	NA	39.3 (35.1–43.7)	NA	12.7 (9.2–17.3)	NA	14.0 (9.5–20.0)	NA	5.2 (3.2–8.4)	NA
No	56.2 (53.4–58.9)	NA	4.7 (3.3–6.8)	NA	3.7 (2.6–5.3)	NA	24.1 (20.4–28.3)	NA	45.1 (41.3–48.9)	NA	9.4 (7.7–11.4)	NA	9.8 (8.2–11.7)	NA	10.1 (8.3–12.4)	NA
**Had drunk alcohol or used drugs before last sexual intercourse**	NA	<0.05	NA	0.35	NA	0.35	NA	0.10	NA	<0.05	NA	0.68	NA	<0.05	NA	0.10
Yes	47.4 (42.0–52.9)	NA	5.7 (3.6–8.9)	NA	2.3 (1.1–5.1)	NA	20.5 (16.2–25.7)	NA	39.3 (33.4–45.5)	NA	10.6 (7.4–15.0)	NA	14.7 (11.0–19.3)	NA	6.4 (3.9–10.5)	NA
No	56.0 (53.1–58.8)	NA	4.7 (3.1–6.9)	NA	3.5 (2.3–5.4)	NA	24.0 (20.4–28.1)	NA	45.1 (41.7–48.6)	NA	9.7 (7.9–11.9)	NA	9.6 (7.6–12.2)	NA	9.6 (7.6–12.1)	NA

**Abbreviations:** CI = confidence interval; IUD = intrauterine device; NA = not applicable.

* Defined as having had sexual intercourse with at least one person during the 3 months before the survey (n = 3,226). Except for any condom use at last sexual intercourse, students reporting only same-sex sexual contact use were excluded; therefore, the analytic sample was restricted to sexually active students who reported having had sexual contact with someone of the opposite sex (n = 2,698). Among sexually active students, excluding those who only had same-sex sexual contact, a total of 93 (3.9%) students answered the pregnancy prevention question “not sure”; findings are not presented for this group.

^†^ Any condom use at last sexual intercourse was measured by a separate item from condoms as the primary method used for preventing pregnancy.

^§^ Weighted estimates.

^¶^ Statistical significance is defined as p<0.05, by chi-square test.

## Discussion

This report provides the most recent nationally representative estimates of condom and contraceptive use among sexually active U.S. high school students. In addition, notable differences in these behaviors by demographic characteristics and sexual risk behaviors are identified that can support implementation of interventions to improve condom and contraceptive use among adolescents most in need. Doing so will help to achieve unintended pregnancy and STD/HIV prevention goals, including reducing disparities by race/ethnicity.

Overall, most (89.7%) sexually active students (excluding those who only reported same-sex sexual contact) used a condom or a primary contraceptive method at last sexual intercourse, yet approximately one fifth (19.8%) reported using withdrawal or some other method only or no condom and no primary contraceptive method. Moreover, prevalence estimates by method type, as well as differences by demographic characteristics and sexual risk behaviors, underscore the importance of meeting the unintended pregnancy and STD/HIV prevention needs of all sexually active high school students.

Only 9.1% of sexually active students (excluding those who only reported same-sex sexual contact) reported having used a condom with a more effective contraceptive method, which is the recommended approach for preventing both unintended pregnancy and STDs/HIV because the most effective forms of contraception confer no STD/HIV protection (*7*–*9*). Although use of condoms alone can prevent both adverse outcomes and was the most prevalent method used, only approximately half of sexually active students reported any condom use at last sexual intercourse, which is concerning given the high risk for STDs among this population (*5*). Moreover, condoms are categorized as a less effective pregnancy prevention method, given that they are associated with a 13.0% pregnancy risk during the first year of typical use (*6*), and prevalence of any highly or moderately effective method use at last sexual intercourse was only 31.4%.

Notable demographic differences in condom and contraceptive use warrant particular attention. Compared with white students, black and Hispanic students had higher prevalence of no pregnancy prevention method use and lower prevalence of highly and moderately effective contraceptive method use. Black students also had lower prevalence of any condom use at last sexual intercourse than white and Hispanic students. On the basis of these findings and the documented racial/ethnic disparities in birth and STD rates among adolescents (*4**,**5*), meeting the unintended pregnancy and STD/HIV prevention needs of black and Hispanic youths is vital. Understanding and addressing structural barriers that might contribute to the observed differences are important next steps. As for grade, differences indicate that younger students are more likely to use condoms, whereas older students are more likely to use an IUD or implant, birth control pills, and condoms with a more effective contraceptive method. Therefore, improving younger adolescents’ knowledge of, comfort with, and access to the most effective methods of pregnancy and STD/HIV prevention is needed. Whereas findings related to race/ethnicity and grade have clear practice implications, patterns by sex might largely reflect reporting differences on the basis of who uses a given method. As compared with female students, the proportion of male students reporting condom use was higher, and the proportion reporting their partners’ use of shot, patch, or ring, and birth control pill use was lower. For the latter female-controlled methods, self-report by females is considered more accurate (*12*).

Finally, differences in condom and contraceptive use by sexual risk behaviors reveal that use of preventive strategies is suboptimal among high school students who engage in those behaviors. The general pattern was that students with a given risk indicator, compared with those without, had lower prevalence of condom use and higher prevalence of using no method of contraception, although not all differences were significant. Such findings might reflect potential disempowerment in sexual interactions (*13*) and the challenge of using condoms correctly and consistently while under the influence of alcohol or drugs (*14*). Because number of partners is an indicator of STD/HIV risk, findings that students with ≥2 recent or ≥4 lifetime partners had lower prevalence of condom use, alone or with a highly or moderately effective contraceptive method, are particularly concerning.

Collectively, these findings from the 2019 YRBS highlight the importance of programmatic efforts that can improve condom and contraceptive use among adolescents. The effectiveness of sexual risk reduction education is well documented (*15*); because of given decreasing attention to condom-related topics in school-based instruction (*16*), efforts to strengthen implementation are warranted. Such education should ensure that highly and moderately effective contraceptive methods are clearly addressed, including in earlier grades (e.g., middle school). Doing so in the context of broader education about health services might be a developmentally appropriate approach.

Engaging directly with communities most affected by unintended pregnancy and STD/HIV can be one strategy to help identify and address social determinants of health that contribute to disparities in condom and contraceptive use. Furthermore, education and clinical services can be delivered through community- and school-based programs tailored to serve young persons most in need. Fostering community–clinic partnerships through youth-serving organizations is one strategy for reaching the most vulnerable adolescents. Such partnerships can help address barriers and improve access to sexual and reproductive health care, either through referral or service integration (*17*).

In addition to access, delivery of comprehensive, client-centered, and adolescent-friendly care by well-trained providers is essential. For example, same-day initiation of long-acting reversible contraception methods (i.e., providing the method during the initial appointment) is a best practice that can facilitate adolescents’ access to these methods (*17*). Another example is provider counseling about condom use with more effective contraceptive methods, which has been associated with adolescents’ use of this prevention strategy (*18*). Integrating unintended pregnancy and STD/HIV prevention in school-, clinic-, and community-based health promotion likely requires explicit attention to individual prevention goals as well as preferences related to the various prevention strategies (*19*).

## Limitations

General limitations for the YRBS are available in the overview report of this supplement (*11*). The findings in this report are subject to at least five additional limitations. First, male students’ report of their female partners’ contraceptive use might not be accurate (*11*). Second, distinguishing the intended purpose of condom use in relation to pregnancy and STD/HIV prevention is not feasible. Although YRBS assesses condom use as a primary method for pregnancy prevention, condom use for STD/HIV prevention is not explicitly measured. Third, condom use with a more effective contraceptive method might be underestimated because respondents could only select one method of pregnancy prevention at last sexual intercourse. Fourth, the estimates for highly and moderately effective contraception could be underestimated if respondents viewed a less effective option (i.e., condoms or withdrawal or some other method) as their primary contraceptive method used at last sexual intercourse. Finally, because the sex of last sex partner is not measured, the analytic sample might include students with same-sex partners at last sexual intercourse for whom pregnancy prevention is not needed. 

## Conclusion

Ongoing national surveillance will remain important to understanding the population-level effects of public health and clinical approaches to preventing unintended pregnancy and STDs/HIV among young persons. To complement these efforts, implementation science and observational research should address unresolved questions (e.g., young men’s role in condom and contraceptive use, barriers and facilitators to integration of pregnancy and STD/HIV prevention, and effective strategies for addressing disparities, including racial/ethnic differences). Taken together, these data can be used to improve condom and contraceptive use for all sexually active adolescents.
